# *Lactobacillus rhamnosus* Probiotic Food as a Tool for Empowerment Across the Value Chain in Africa

**DOI:** 10.3389/fmicb.2018.01501

**Published:** 2018-07-10

**Authors:** Nieke Westerik, Remco Kort, Wilbert Sybesma, Gregor Reid

**Affiliations:** ^1^Yoba for Life foundation, Amsterdam, Netherlands; ^2^Department of Molecular Cell Biology, VU University Amsterdam, Amsterdam, Netherlands; ^3^Department of Microbiology and Systems Biology, Netherlands Organization for Applied Scientific Research (TNO), Zeist, Netherlands; ^4^Canadian R&D Centre for Human Microbiome and Probiotics, Lawson Health Research Institute, London, ON, Canada; ^5^Departments of Microbiology and Immunology, Surgery, Western University, London, ON, Canada

**Keywords:** probiotics, fermented food, Africa, microenterprises, *Lactobacillus rhamnosus*

## Abstract

Perhaps by serendipity, but *Lactobacillus rhamnosus* has emerged from the 1980s as the most researched probiotic species. The many attributes of the two main probiotic strains of the species, *L. rhamnosus* GG and GR-1, have made them suitable for applications to developing countries in Africa and beyond. Their use with a *Streptococcus thermophilus* starter strain C106, in the fermentation of milk, millet, and juices has provided a means to reach over 250,000 consumers of the first probiotic food on the continent. The social and economical implications for this translational research are significant, and especially pertinent for people living in poverty, with malnutrition and exposure to environmental toxins and infectious diseases including HIV and malaria. This example of probiotic applications illustrates the power of microbes in positively impacting the lives of women, men, and children, right across the food value chain.

## Introduction

The appreciation that certain bacterial species can provide benefits to the human body dates back many years. This has led to the term probiotics, defined as live microorganisms that when administered in adequate amounts, confer a health benefit on the host (Hill et al., 2014). The application of a living microbe to humans has primarily involved oral intake in a food or dried supplement form. In most countries, if only generic and non-disease claims are made, the product does not need to be registered as a drug. This has allowed food companies and small to medium sized enterprises the ability to enter the rapidly growing market for probiotics, where profit margins are a fraction of those in the pharmaceutical industry. The perceived inability to protect intellectual property around probiotic applications, has so far kept big pharma from developing probiotic drugs.

The net effect is that the probiotics mostly documented scientifically and clinically, have been with food and supplement applications in mind. Furthermore, they are microbial species with a history of safe use, which again is due to their inclusion in a variety of foods. This might explain, together with their suitability for large scale cultivation, why the most studied *Lactobacillus* species for human application is *Lactobacillus rhamnosus*. This species can be recovered from some fermented foods and the intestinal and vaginal tracts, and strains appear to possess a number of interesting characteristics suitable for use in humans (Segers and Lebeer, 2014).

## Why *L. rhamnosus* As a Probiotic?

Arguably, there are two main approaches to selecting a strain to be probiotic. The first is to use a species normally abundant at a site, and simply replenish or boost the total count of that species in subjects whose microbiota has shifted to being dysbiotic. The second approach is to select a strain(s) that has specific properties to counter an ailment.

In the case of *L. rhamnosus* GG, isolated in 1983 in Boston, it was quickly commercialized with the idea that it could provide health benefits, with the first paper suggesting this published in 1993 (Goldin et al., 1992). Since then, of course, it has become the most researched probiotic strain, primarily for gut health, with over 900 publications on pubmed.

In the case of *L. rhamnosus* GR-1, the second most scientifically documented *L. rhamnosus* probiotic, it was discovered in 1981 in Kingston, Ontario, and not commercialized until over 20 years later, in order to acquire as much data on the strain as possible. While it shares some genomic properties with *L. rhamnosus* GG (Petrova et al., 2018), its main attributes appear to be the ability to counter pathogenic bacteria and fungi in the urogenital tract (Reid, 2017). The strain is available for intravaginal administration in Croatia, but due to such applications coming under a drug category in many regions of the world, thereby requiring huge development costs for approval, it has not been approved in other countries for such use.

As urogenital infections originate from microbes ascending from the rectum, studies were performed to show that oral intake of GR-1 provided benefits to the urogenital tract as well as the gut and respiratory tract (Baroja et al., 2007; Koyama et al., 2010; Reid, 2010). Since for a long time the female urogenital tract was not a popular research-funded area and lacked industrial investors, the strain was not sought by other groups for clinical studies until around the year 2000. Studies thereafter have shown that in combination with *L. reuteri* RC-14, there is improved treatment of vaginal infections (Reid et al., 2001; Macklaim et al., 2015), reduced carriage of group B streptococci in pregnant women (Ho et al., 2016), and an ability to prevent recurrent urinary tract infections in post-menopausal women (Beerepoot et al., 2012). These are extremely common conditions that result in millions of antibiotic prescriptions each year. The continuing increase of antibiotic resistance worldwide is alarming and the side effects of antibiotic use on people’s microbiome and resilience are worrying. Therefore, well-tolerated, highly effective therapeutic alternatives are urgently needed. While one study showed that *L. rhamnosus* GR-1 and *L. reuteri* RC-14 taken daily could reduce the incidence of UTI over 1 year by more than 50% (Beerepoot et al., 2012), even a 25% reduction in antibiotics would have significant implications for women’s health and the healthcare system, but also for babies at risk because of the urogenital colonization of pathogens.

In terms of safety, in a study of the genomes of 17 *L. rhamnosus* strains, it was shown that isolates from the blood of patients with bacteremia were distinguishable from *L*. *rhamnosus* GG (Nissilä et al., 2017). This proved that the GG strain was not the cause of bacteremia, however, the study did not examine properties that could make it, or the other strains, act in a probiotic manner. No safety concerns have been raised for *L. rhamnosus* GR-1, even when used in inflammatory bowel disease and HIV patients (Baroja et al., 2007; Reid, 2011). Such safety assessments are important when proposing to administer probiotic *Lactobacillus* strains to populations that may contain people defined as high-risk, because of immune suppression, HIV infection, or being severely malnourished (Sanders et al., 2016). This makes it vital that the *L. rhamnosus* strains used in these settings are prepared to stringent microbiological standards.

Another study of 40 *L. rhamnosus* strains described 2,164 core genes, out of the pan genome of 4,711 genes. With an average genome size is 3 ± 0.2 Mb, the *L. rhamnosus* genomes are among the largest for the *Lactobacillus* group with the mean percentage G + C content comparable to *L. casei* species (46.6% compared with 46.3%), suggestive of a strain capable of adaptation (Ceapa et al., 2016). Analysis shows the species has the capacity to transport and metabolize carbohydrates, produce muramidases p40 and p75 to improve epithelial layer integrity and repair, and in some strains to produce bacteriocins. These are traits that are useful for probiotic strains with intestinal applications. Specific properties attributed to promotion of health by the GG strain include the production of pili, which likely facilitate adhesion to the intestinal mucosa and immunomodulation (Lebeer et al., 2012; Tytgat et al., 2016). However, the encoding genes are located at a genetically unstable island in the genome and may get lost upon repetitive back-slopping (Sybesma et al., 2013; Kort et al., 2015). Other research has also shown the ability of the GG and GR-1 strains to bind aflatoxins (Ahlberg et al., 2015; Nduti et al., 2016), as further commented below.

## The Rationale For Applications In Africa

The regulatory stipulations that make the drug route of approvals so costly and time-consuming, were a major driver in the use of the GG and GR-1 strains in foods and supplements, with minimal claims. Yet, it is clear that the strains in many occasions do counter diseases, as discussed later, even if companies are not allowed to convey that message to the consumer. But, for application to Africa, cost and sustainability are more important reasons to make locally produced yogurt as opposed to tablets. At production sites, 100 ml yogurt containing an estimated 2.5 × 10^9^ CFU of *L. rhamnosus* GG or GR-1 costs only around US$0.14. This takes advantage of the growth of the strains in the milk, with up to 100 L made per batch, whereas tablets and capsules of these strains would have to be imported and properly stored, which makes them more expensive. Plus, people might only take them if they felt sick, whereas yogurt is part of daily sustenance.

The use of a milk delivery vehicle was based on the history of *L. rhamnosus* in dairy products (Lazzi et al., 2014), the ease of production of yogurt, and the long history of fermented foods in Africa (Franz et al., 2014). In response to an appeal for assistance in the AIDS epidemic, a program called Western Heads East was established, and in 2004, local mothers in Mwanza, Tanzania were taught how to produce Africa’s first probiotic fermented food in form a fermented milk, using *L. rhamnosus* GR-1 as the supplemental culture.

**FIGURE 1:**
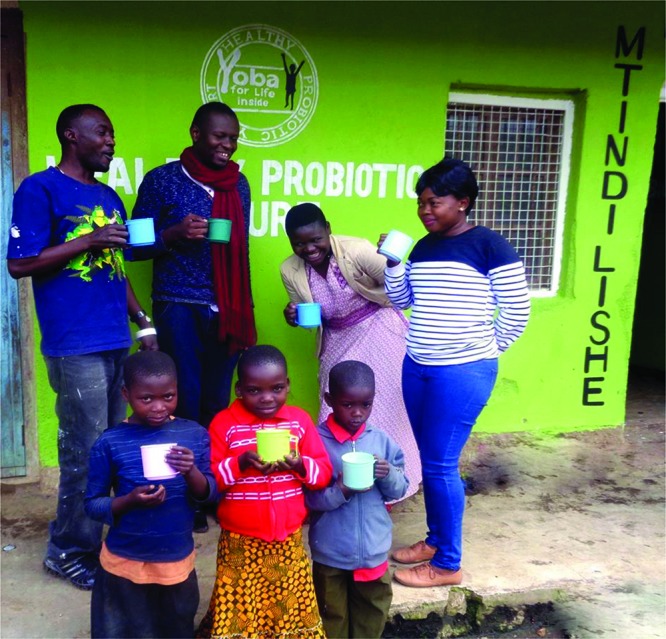
People enjoying Yoba outside a production unit in Tanzania.

This proved to be a societal breakthrough perhaps even more than a scientific one, in that it provided people living in a poor community the ability to produce a health-promoting food and make a living doing it. While many challenges arose, from how the probiotic strain was propagated and added to the starter cultures in milk, to educating consumers about probiotics and fermented food, and establishing satisfactory, reproducible yogurt (Reid et al., 2013), it was the catalyst for change resulting today in an infrastructure of over 220 small scale production units producing and selling their own probiotic yogurts in Tanzania, Uganda, and Kenya (Reid et al., 2013) (**Figure 1**).

With high rates of malnutrition, maternal, and infant mortality, and poverty, Tanzania was in 2004 a country facing significant challenges. Humanitarian aid and structural development programs are provided by many charitable organizations and developed countries, with perhaps the most important being access to clean water and basic health services including for immune-compromised people suffering from HIV/AIDS. Expensive peanut-based, such as Plumpy′nut^®^ (produced in France and shipped to developing countries (with price to consumer of around US$0.40 each) (Ali et al., 2013), or foods that support gut microbial diversity and reduce malnutrition developed in resource-rich countries may be effective, but will require significant donations to pay for them and thus lack sustainability, and are not so far enabling to the communities that need them.

In sustainable development, it is vital that interventions empower local people with tools that allow them to take control of their situation. This was the rationale for our approach in East Africa. Historically, fermentation, particularly of cereals, involves back-slopping and does not incorporate probiotic strains. In Uganda, poor sanitation conditions, insufficient heat processing and inadequate storage conditions of cereal porridges sometimes used for infant feeding can contribute to contamination with, and proliferation of diarrhea causing pathogens (Steinkraus, 1995). For rural children, porridge is boiled and immediately consumed (at home or at school).

**FIGURE 2:**
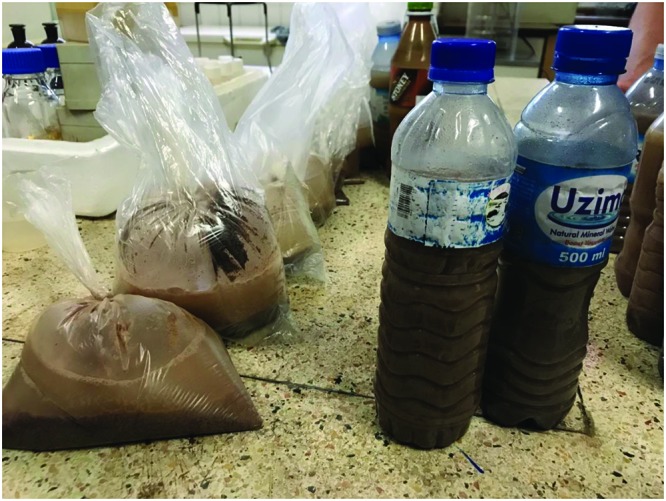
Samples of Obushera (fermented millet) sold in the streets of Kampala, Uganda, and shown in Dr. Ivan Mukisa’s lab at Makerere University, to be contaminated.

A recent study (Byakika unpublished) indicates that about 50% of traditionally produced commercial *Obushera* on the market in Kampala does not meet the microbial safety requirements. Furthermore, it is produced with very poor hygiene conditions sold in pre-used plastic bottles or in plastic bags that would not be acceptable in developed countries, as shown in (**Figure 2**). It tends to be purchased by the adult working class.

While the process of fermentation can overcome growth of pathogens to some extent, and not all street-sold products will cause diarrhea, contamination by bacterial and fungal pathogens can easily occur. Clearly hygienic preparatory and packaging methods are important, as are the strains being used to ferment the food and degrade aflatoxins in the case of cereal-based fermented foods. This latter issue is particularly important in a region of the world where aflatoxin poisoning or contamination at least, is common and associated with developmental impairment in children, and cancer in adults (Kaaya and Kyamuhangire, 2006; Kitya et al., 2010). In addition, these carcinogenic substances produced by molds in poorly stored food, have also found to be a precursor for stunting (Kitya et al., 2010). Aflatoxins are found in alarmingly high levels in commonly consumed foods in Uganda, most especially in ground nuts and maize (Kaaya et al., 2005).

## Supporting Evidence

A small intervention study using a fermented milk containing *L. rhamnosus* GR-1 along with *Streptococcus thermophilus* and *Weissella cibaria* NN20 consumed daily, found that aflatoxin concentrations in urine were significantly lower than baseline, but increased in the milk control group (Nduti et al., 2016). The mechanism reported included binding of the lactic acid bacteria to the aflatoxins. *L. rhamnosus* GG has been shown to bind aflatoxin B1, thereby reducing its absorption into the intestine, and aflatoxin associated pathogenicity including stunting (Haskard et al., 2000; Gratz et al., 2006). Studies in our group have shown that *L. rhamnosus* GG actually degrades aflatoxins (unpublished). While contamination of maize and peanuts would not be expected to pass food safety checks in countries like Canada, aflatoxin contamination is highly prevalent in the developing world. So, the ability of *L. rhamnosus* to bind to, and degrade these compounds is an invaluable trait.

This attribute of *L. rhamnosus* GG, along with many other documented probiotic effects it conveys, was a motivating factor in it being selected for use in African populations. With its patent protection ended, the strain was isolated and renamed Yoba 2012, then packaged with *S. thermophilus* C106 in a sachet that can be used to produce 100 L of probiotic fermented food (Kort et al., 2015; Sybesma et al., 2015). It is currently distributed across Uganda and Tanzania where local people produce probiotic fermented milk for distribution to over 150,000 people. Over 1.6 million L have been produced in Uganda alone, during the years 2016-18. In a similar way in Kenya and the Mwanza region of Tanzania, the *L. rhamnosus* GR-1 strain in combination with *S. thermophilus* C106 is used to make the probiotic fermented food (Reid et al., 2018) reaching over 100,000 consumers weekly.

The prevention and reduction of the duration of diarrhea (Vanderhoof et al., 1999; Basu et al., 2007) were motivational factors for *L. rhamnosus* GG being used in Uganda and Tanzania, and there is no reason to think that such benefits would not be conveyed to African populations, where the disease is responsible for 13% of the deaths of children between 1 and 59 months of age (Uganda Bureau of Statistics [UBOS] and ICF, 2017). In Uganda, 29% of the children <5 years of age has been found to be stunted, and 4% wasted (Uganda Bureau of Statistics [UBOS] and ICF, 2017). It was nevertheless important to perform further studies. While these may not be as large in size as preferred, nor able to verify mechanisms of action, they support the ultimate outcome, namely of assessing how consumption affected lives.

In Uganda, an open label primary school study of 245 children, showed significant reduction in skin allergies (allergic dermatitis, pyoderma, tinea capitis, and tinea corporis), and reported reduction in diarrhea following 5 weeks of Monday–Friday consumption of 100 ml Yoba probiotic yogurt containing *L. rhamnosus* GG (Westerik et al. unpublished). This alleviation of skin conditions is supported by studies showing that *L. rhamnosus* GG greatly reduced eczema and allergic reactions (Viljanen et al., 2005; Berni Canani et al., 2017).

In another school study, 4 weeks of Monday–Friday consumption of Fiti probiotic yogurt containing *L. rhamnosus* GR-1 or milk was well tolerated and showed a trend to lowering uptake of heavy metals, but it was not statistically significant (Bisanz et al., 2014). Whereas, taken daily for 3 months by pregnant women, it resulted in 75% less uptake of arsenic and 36% less uptake of mercury known to be contaminants present in fish from Lake Victoria and possibly from other foods. The GR-1 and GG strains do not have the *mer*R gene, regulating the organomercurial resistance system, but they do bind to a number of heavy metals, aflatoxins and environmental chemicals, making them useful in settings where people are exposed to such compounds. This is not restricted to people living on Lake Victoria. Rather, the Great Lakes of North America and food supplies around the world can be contaminated with toxins. If such adsorption could be reduced simply by consumption of probiotic *L. rhamnosus*, one would expect long term that fewer cases of environmental-related diseases might occur. Proof would require large longitudinal studies, or at least before-and-after sampling of people consuming contaminated food and water, and who take *L. rhamnosus* GR-1 or GG, or indeed other probiotic strains with such binding activity.

Studies have shown that the Fiti yogurt containing the *L. rhamnosus* GR-1, can also reduce skin rashes and diarrhea, as well as decrease fatigue in HIV patients (Anukam et al., 2008; Reid, 2010). As with *L. rhamnosus* GG being safe for use in HIV patients (Salminen et al., 2004), the GR-1 strain in yogurt is also safe and has resulted in some patients presenting with intestinal benefits and increased CD4 counts, indicating an enhanced immune system (Irvine et al., 2010; Hummelen et al., 2011; Hemsworth et al., 2012). Notably, these latter studies are not claiming consistent population-wide effects for HIV subjects, since some patients do not show this effect (Hummelen et al., 2011), but there were certainly people who responded extremely well to the intervention, unlikely to be simply due to a placebo effect. In a number of cases, the patients were about to be given anti-retroviral therapy, but the increase in CD4 count led to them not being prescribed (subsequently inclusion criteria has changed). From an educational perspective, there was clearly a need to explain to patients and caregivers that probiotics are not a substitute for drug treatment, irrespective of how well people felt. Mechanistically, the effect was presumably induced through host immune modulation, a characteristic known to be conveyed by the GR-1 (Kim et al., 2006). Enhancement of gut barrier function may also have enhanced nutritional status and indirectly increased T cell counts.

## Reducing Post-Harvest Crop Losses

Climactic conditions can have major effects on milk and crop production in East Africa. This makes it important to find alternatives to milk as a carrier for the probiotics. Two good options are fruits and cereals. Both suffer post-harvest losses that have major economic and societal implications. Cereals are part of the staple diet, and even included in school feeding programs, primarily in the form of maize porridge. Experiments performed in Canada, showed that probiotic fermented juices and millet could be created using the Fiti sachets that are used in East Africa (Di Stefano et al., 2017; Reid, 2017; White and Hekmat, 2018). Fermented millet and maize has also been produced using the Yoba sachets in Uganda. Community kitchens are being taught how to replicate these processes to expand the options for consumers. This illustrates the flexibility of the *L. rhamnosus* strains together with the *S. thermophilus* C106 and provides an excellent example of this combination of species being exploited for local food and health-related applications. Reduction in post-harvest losses would have major economical and social implications, providing more income for street-sellers and fermented food producers.

The ability of *L. rhamnosus* strains to produce vitamins, antimicrobial activity and to detoxify carcinogens, make them very useful to prevent post-harvest crop losses through fermentation.

Further studies are underway in Africa to document the benefits of the probiotic fermented food. A series of case studies illustrated the personal life-changing effects of the introduction of Yoba and Fiti to regions of East Africa on wealth and health^1^.

## Concluding Remarks

*Lactobacillus rhamnosus* species has a long history of being used in fermentation, and it possesses a genome that allows it to adapt to a range of environments, including the human gastrointestinal and urogenital tracts. The ability of two strains of this species to produce ferment foods has resulted in them being part of a large sustainable structural development effort in East Africa. Combined with starter culture *S. thermophilus* C106, packaged in a sachet affordable to people in resource poor areas of the world, the resultant products are highly nutritious, confer a range of health enhancing properties, and form the basis of micro-enterprises bringing societal benefits across the value chain. It is perhaps the ultimate translation of naturally occurring bacteria to humanity.

## Author Contributions

All authors listed have made a substantial, direct and intellectual contribution to the work, and approved it for publication.

## Conflict of Interest Statement

The authors declare that the research was conducted in the absence of any commercial or financial relationships that could be construed as a potential conflict of interest.

## Footnotes

1 http://www.yoba4life.org/
